# CYP2C19 Polymorphism and Platelet Aggregation‐Associated Risks in Atrial Fibrillation Patients Undergoing PCI

**DOI:** 10.1111/cts.70598

**Published:** 2026-05-19

**Authors:** Diona Gjermeni, Sofia Szabó, Viktoria Anfang, Hannah Vetter, Carina Juelch, Svenja Rademacher, Tian Li, Stefan Leggewie, David Hesselbarth, Daniel Duerschmied, Dietmar Trenk, Dirk Westermann, Christoph B. Olivier

**Affiliations:** ^1^ Department of Cardiology and Angiology, University Heart Center Freiburg—Bad Krozingen, Faculty of Medicine University of Freiburg Freiburg Germany; ^2^ Institute of Human Genetics, Medical Center ‐ University of Freiburg, Freiburg, Faculty of Medicine University of Freiburg Freiburg Germany; ^3^ Department of Cardiology, Haemostaseology and Medical Intensive Care, Medical Center Mannheim, Medical Faculty Mannheim Heidelberg University Heidelberg Germany; ^4^ European Center for AngioScience (ECAS) and German Center for Cardiovascular Research (DZHK) Partner Site Heidelberg/Mannheim Mannheim Germany

**Keywords:** atrial fibrillation, cytochrome P450 2C19, percutaneous coronary intervention, platelet aggregation, platelet function testing

## Abstract

*CYP2C19* genetic polymorphisms impact the antiplatelet effect of clopidogrel and associate with ischemic and bleeding risk in patients undergoing percutaneous coronary intervention (PCI). This study aimed to evaluate the association of *CYP2C19* and platelet reactivity (PR) with these risks, in atrial fibrillation (AF) patients undergoing PCI, treated with an oral anticoagulation (OAC) and clopidogrel. This two‐center prospective cohort study included patients with AF and OAC undergoing PCI. Carriers of ≥ 1 loss‐of‐function (LOF) allele were classified as poor/intermediate metabolizers (PM/IM), whereas carriers of ≥ 1 gain‐of‐function allele without LOF alleles were classified as rapid metabolizers (RM). PR was assessed by thromboelastography (TEG) and/or multiple electrode aggregometry (MEA). The primary outcome included death, MI, or stroke at 6 months ±2 weeks; the secondary outcome consisted of non‐major clinically relevant (NMCR) or major bleeding. Among 283 patients (median age 78 years; 72% male), 73 (26%) were PM/IM, 108 (38%) were NM, and 102 (36%) were RM. PM/IM status was not significantly associated with the primary ischemic outcome (PM/IM: 6 [8.2%] vs. NM + RM: 16 [7.6%], *p* = 0.869), but any bleeding rates were numerically lower. While there was a trend for association of high platelet reactivity (HPR) with the ischemic outcome (OR 2.005 [95% CI 0.820–4.902], *p* = 0.127), low platelet reactivity (LPR) was associated with major bleeding (OR 2.646 [95% CI 1.075–6.509], *p* = 0.034). PM/IM status did not detect an increased ischemic risk but might, however, protect from bleeding risk in AF patients undergoing PCI. HPR might indicate a higher ischemic risk, while LPR was associated with major bleeding.

## Study Highlights

- What is the current knowledge on the topic?
  ○ Both cytochrome polymorphism and ex vivo platelet function associate with outcomes in patients undergoing PCI. However, this relationship has not been confirmed in patients undergoing PCI with additional AF and an OAC, who present with substantially increased bleeding and ischemic risk.
- What question did this study address?
  ○ Do CYP2C19 polymorphisms and platelet aggregation biomarkers associate with clinical risk factors and outcomes in atrial fibrillation patients undergoing PCI, and can they help guide antithrombotic treatment?
- What does this study add to our knowledge?
  ○ In patients with AF undergoing PCI, poor/intermediate metabolizer status may confer a protective effect against bleeding events. High platelet reactivity as assessed by TEG and/or MEA might indicate a higher ischemic risk, whereas low platelet reactivity was significantly associated with major bleedings.
- How might this change clinical pharmacology or translational science?
  ○ Further research to better define the role of genetic testing and platelet function testing in advancing personalized treatment strategies in cardiology is needed. These approaches may improve risk stratification and help identify patients who could benefit from tailored escalation or de‐escalation strategies.

## 1 Background

Atrial fibrillation (AF) is the most common sustained supraventricular arrhythmia affecting approximately 11 million people in Europe and its incidence is increasing with age [1]. Notably, 70%–80% of AF patients face a moderate to high risk of cerebral thromboembolic complications and guidelines recommend oral anticoagulation (OAC) as standard treatment to mitigate this risk [2]. About 20%–30% of AF patients present with concomitant coronary artery disease (CAD). In the first post‐PCI week or during hospitalization antithrombotic treatment (ATT) includes a direct oral anticoagulant (DOAC), acetylsalicylic acid (ASA), and a P2Y12 inhibitor (most likely clopidogrel) [2, 3]. Subsequently, guidelines suggest transitioning to dual ATT with a DOAC and clopidogrel for 6–12 months [2]. After omitting ASA, patients with variable on clopidogrel platelet reactivity (PR) based on *CYP2C19* polymorphism may face post‐PCI higher ischemic or bleeding risk. While genotype and platelet function testing (PFT)‐guided ATT have been investigated in patients undergoing PCI [4, 5], studies in patients with additional AF are lacking. Thromboelastography (TEG) and multiple electrode aggregometry (MEA) are widely used to assess PR. High and low platelet reactivity (HPR/LPR) associate respectively with increased ischemic and bleeding risk in PCI patients. In particular, HPR rates in PCI patients range from 21%–50% when assessed by TEG [5].

Clopidogrel is a prodrug that is metabolized and activated by cytochrome *CYP2C19* P450. Loss of function (LOF) allele *2 is associated with an increased ischemic risk at 5 years after PCI [6]. In addition, genotype‐guided therapy associates with 28% lower bleeding risk after PCI in patients with rapid metabolizer status [7, 8]. Current consortium guidelines and international consensus document [9, 10] suggest genotyping of *CYP2C19* in high‐risk patients when selecting antiplatelet treatment after PCI. Thus, *CYP2C19* genotyping and PFT are crucial to lead the way toward the much‐needed personalized medicine.

Aim of this study is to evaluate the association of *CYP2C19* polymorphism and PR with ischemic and bleeding risk in patients with AF undergoing PCI treated with clopidogrel and an OAC.

## 2 Methods

### 2.1 Study Design and Population

Data were pooled from two two‐center prospective observational cohort pilot studies. Between May 2020 and October 2022, 283 patients with AF undergoing PCI were enrolled. *CYP2C19* genotyping was performed as an exploratory marker analysis within two studies primarily designed to assess platelet function using multiple electrode aggregometry (MEA) and/or thromboelastography (TEG) in patients with AF undergoing PCI. These studies were approved by the Ethics Committee of the Albert‐Ludwigs‐University Freiburg, Germany (registry numbers 194/20 and 21–1146) and were registered in the German Clinical Trials Register (DRKS00021212 [11] and DRKS00024509 [12]). Since at the time of studies desing, no studies considering AF patients were available, respective sample size calculations were based on studies that assessed the association of platelet function with clinical outcomes only in patients undergoing PCI [13, 14]. Combined TEG/MEA measurements lack in the totality of the cohort, as the analyzed sample represents the combined pool from the two previous studies, which were conducted and funded separately [15, 16].

All patients provided written consent and were excluded if they had a history of stent thrombosis, use of GPIIb/IIIa in the last 24 h, or intake of a P2Y12 inhibitor other than clopidogrel in the last 7 days. Inclusion and exclusion criteria are listed in the supplement (Table S1). Patients were grouped according to the *CYP2C19* genotype and their PR status.

### 2.2 Percutaneous Coronary Intervention

Patients with ST‐elevation myocardial infarction, non‐ST‐elevation myocardial infarction (NSTEMI), or elective PCI were included. Arterial access was at the discretion of the interventionalist. All patients received at least one drug‐eluting stent (DES) along with 75 mg clopidogrel and OAC post‐procedure. Discharge from hospital and management of in‐hospital complications were performed per standard of care.

### 2.3 Blood Samples, Genotyping and Platelet Aggregometry

Venous blood was collected once on day 1 to 3 after PCI, using a 21G butterfly needle (Safety‐Multifly‐Set, Sarstedt, Nümbrecht, Germany) and anticoagulated to a final concentration of > 15 μg/mL r‐hirudin (SARSTEDT Monovetten, Nümbrecht, Germany). Clopidogrel was administered orally at 7 AM with blood drawn at least 1 h later. A 2.7 mL EDTA S‐Monovette K3 (preparation concentration: 1.6 mg/mL blood, Sarstedt, Nümbrecht, Germany) was preserved for subsequent genotyping and stored at −20°C. Genomic DNA was extracted from leukocytes and the two allele loci CYP2C19*2 c.681G>A, p. (Pro227) (rs4244285) and CYP2C19*17 c.‐806C>T (rs12248560) were analyzed by Sanger sequencing using standard methods for polymerase chain reaction (PCR) with an ABI 3500XL DNA sequencer (Applied Biosystem, Foster City, USA). The subtype CYPC19*2 is characterized by the homo‐ or heterozygous presence of the base pairs guanine (G) and the loss‐of‐function allele alanine (A), while CYP2C19*17 is represented by the base pairs cytosine (C) and the gain‐of‐function allele thymine (T). Patients were grouped in three groups according to *CYP2C19* polymorphism status. Carriers ≥ 1 loss‐of‐ function (LOF) allele (CYP2C19*2) were classified as poor/intermediate metabolizers (PM/IM) and carriers of ≥ 1 gain‐of‐function (GOF) allele (CYP2C19*17) in the absence of a (LOF allele) were classified as rapid metabolizers (RM). Normal metabolizers (NM) were defined by the absence of CYP2C19*2 or *17 alleles [9].

Multiple electrode aggregometry (MEA, Roche Diagnostics, Risch‐Rotkreuz, Switzerland) and Thromboelastography (TEG 6 s Hemostasis Analyzer, Haemonetics Corp., Boston, MA, USA) were performed on day 1 to 3 after PCI. MEA measurement was performed within 3 h after blood draw. For MEA, whole blood was stimulated with adenosine diphosphate (ADP; final concentration 6.4 μM) or thrombin receptor activating peptide‐6 (TRAP; final concentration 32 μM). Due to the strongly divergent results obtained with MEA and TEG, we defined HPR and LPR‐status based on the upper or lower quartile of platelet aggregation values. HPR_MEA_ was defined as values of ADP‐induced aggregation at the upper quartile of the area under the aggregation curve [AUC] < 17.5 U and LPR_MEA_ was defined as the lower quartile with AUC < 6.7 U. Reference values for TRAP‐induced platelet aggregation, were 94–156 U and were based on the expert consensus on platelet function and genetic testing [4].

To perform TEG, approximately 400 μL blood was automatically aspirated into the testing area. Afterwards, 2 μM ADP was used as a reagent for measuring platelet function, and kaolin with heparinase (concentration > 1800 IU/mL) was used for assessing overall aggregability. The maximum amplitude (MA, [mm]) was determined to describe the maximum clot strength. HPR_TEG_ was defined as the upper quartile of MA_ADP_ > 58.1 mm and LRP_TEG_ as the lower quartile with MA_ADP_ < 39.9 mm after stimulation with ADP. Kaolin‐activated channel HKH‐channel was used for MA_Thrombin_. Reference values of MA_Thrombin_ (53–68 mm) were based on the expert consensus [4].

### 2.4 Study Outcomes and Follow‐Up

The primary composite ischemic outcome consisted of time to all‐cause mortality, myocardial infarction, or stroke within 6 months after PCI [17]. The secondary outcome included time to non‐major but clinically relevant bleeding (NMCR) or major bleeding according to International Society on Thrombosis and Hemostasis (ISTH) [18, 19]. Follow‐up was conducted through telephone interviews at 6 months ±2 weeks with event verification through further documentation such as discharge letters, angiography reports, or death certificates. The event dossier was submitted to two independent physician reviewers. Significant discrepancies were resolved by a board‐certified cardiologist who was blinded to PFT results. Patients were divided into 3 groups based on their *CYP2C19* genotype as poor/Intermediate, normal or rapid metabolizer [4] whereas for assessing PR into 4 groups that were compared as follows: HPR versus non‐HPR, LPR versus non‐LPR.

### 2.5 Statistical Considerations

Categorical variables were presented as number and proportion and continuous variables as median with interquartile range (IQR). Mann–Whitney‐*U* test was used to compare the median distribution while Chi quadrat test to compare proportions between groups. For groups with < 15 events, Fisher's exact test was used.

The number of patients free from ischemic, thromboembolic, and bleeding events until follow up was assessed through Cox‐regression analyzes. Univariate logistic regression was used to determine the association of variables with the clinical outcomes. All tests were 2‐tailed and *p*‐values ≤ 0.05 were considered statistically significant. Data were analyzed with Prism 9.2.0 (GraphPad Software, La Jolla, California, USA) and SPSS 27.0.0.1 (SPSS Inc., Chicago, Illinois, USA).

## 3 Results

### 3.1 Baseline Characteristics and Periprocedural Medication

The median age was 78 years (interquartile range, IQR 72–82) and 204 (72%) patients were of male sex. The median CHA_2_DS_2_‐VASc score was 5 (IQR 4–6) and the median HAS‐BLED score was 3 (IQR 3–4). 140 (50%) patients had a history of PCI, and 59 (21%) patients had a history of myocardial infarction (MI). 73 (26%) presented with an acute coronary syndrome (ACS) (Table 1). All patients received clopidogrel prior to stent implantation: 47 (17%) received 300 mg clopidogrel, 174 (61%) received 600 mg, and 61 (22%) were on maintenance therapy (Table S2). At discharge, 281 (99%) patients were treated with a DOAC primarily rivaroxaban (115 [41%]) and apixaban (99 [35%]) (Table S2). Two patients did not receive oral anticoagulation due to gastrointestinal bleeding with subsequent left atrial appendage (LAA) occlusion and hemodialysis.

**TABLE 1:** Baseline and procedural characteristics of the population.

Baseline characteristics	Total		PM/IM		NM		RM		*p*
	*n* = 283		*n* = 73	26%	*n* = 108	38%	*n* = 102	36%	
Demographics									
Male sex	204	(72%)	62	85%	75	69%	67	66%	0.015
Age [years]	78	[72–82]	79	[71–83]	77	[72–82]	79	[72–82]	0.178
Body mass index [kg/m^2^]	26	[24–30]	26	[24–29]	27	[24–31]	26	[25–31]	0.738
Medical history									
Type of AF									0.922
Paroxysmal	171	61%	43	59%	63	58%	65	63%	0.693
Persistent	60	21%	17	23%	24	22%	19	19%	0.718
Permanent	52	18%	13	18%	21	20%	18	18%	0.935
CHA_2_DS_2_‐VAS_C_	5	[4–6]	5	[4–6]	5	[4–6]	5	[4–6]	0.651
Previous MI	59	21%	14	19%	19	18%	26	25%	0.341
Stroke/TIA	42	15%	14	19%	13	12%	15	15%	0.415
Gastrointestinal bleeding	15	5%	5	7%	6	6%	4	4%	0.688
Intracranial bleeding	6	2%	1	1%	5	5%	0	0%	0.058
Previous PCI	140	50%	41	56%	50	46%	49	48%	0.401
CVD risk factors									
Arterial hypertension	248	88%	65	89%	91	84%	92	90%	0.389
Diabetes mellitus	99	35%	25	34%	42	39%	32	31%	0.515
History of smoking	22	8%	8	11%	6	6%	8	8%	0.731
Procedural characteristics									
Index event of PCI									0.793
Elective	210	74%	55	75%	82	76%	73	72%	0.746
Thrombocytes [10^3^/μL]	207	[174–266]	212	[177–253]	215	[180–277]	200	[168–252]	0.545
IPF [%]	4	[3–5]	4	[3–6]	4	[3–5]	4	[3–5]	0.472

*Note:* The values are *n* (%) or median (interquartile range, IQR).

Abbreviations: AF, atrial fibrillation; CVD, cardiovascular; IM, intermediate metabolizer; IPF, Immature platelet fraction; MI, myocardial infarction; NM, normal metabolizer; PCI, percutaneous coronary intervention; PM, poor metabolizer; RM, rapid metabolizer; TIA, transient ischemic attack.

### 3.2 *CYP2C19* Polymorphism

Genotyping was performed in all patients included in the study. 73 (26%) patients were carriers of at least one LOF (CYP2C19*2) allele and thus had a PM/IM status. Of these, 8 (3%) patients were PM and 65 (23%) patients were IM, whereas 108 (38%) patients were NM and 102 (36%) were RM.

PM/IM group had a higher proportion of males (62 [85%]) compared with the NM (75 [69%]) and RM (67 [66%]), *p* = 0.015, (Table 1). No other statistically significant differences in the baseline characteristics were observed among *CYP2C19* genotype groups (Table 1).

### 3.3 Platelet Aggregation Status

At least one of PFT was performed on all patients included in this study. In 156 (55%) patients, platelet aggregation was assessed by MEA, in 174 (61%) patients by TEG, whereas 39 (14%) patients received both TEG and MEA measurements.

**FIGURE 1:**
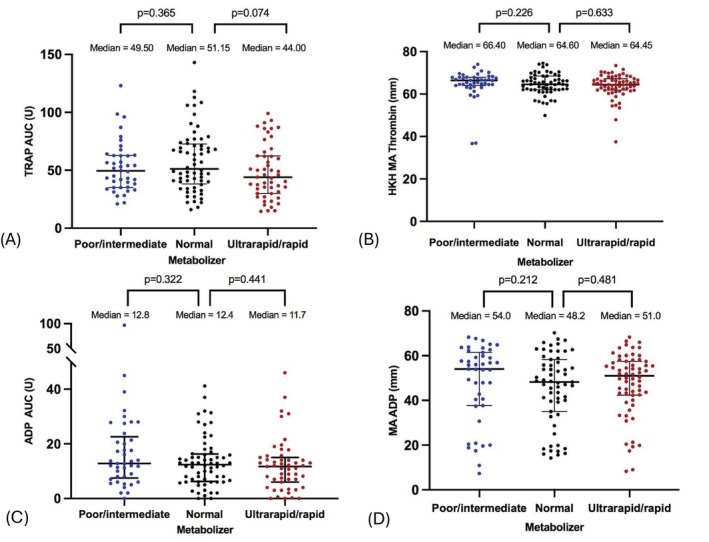
Overall platelet aggregation and on clopidogrel platelet reactivity as assessed by MEA (A, C) and TEG (B, D) for the different *CYP2C19* polymorphisms. ADP AUC, adenosine diphosphate–area under the curve; IM, intermediate metabolizer; MA, maximal amplitude; NM, normal metabolizer; PM, poor metabolizer; RM, rapid metabolizer.

When using the standard cut‐offs, 2 (1%) patients had HPR by MEA [11] whereas 101 (61%) had HPR by TEG [12, 20]. Because of the divergent HPR rates detected by these methods, we considered the upper and lower quartile of platelet aggregation values (TEG and/or MEA) to redefine HPR and LPR. We thus detected HPR in 76 (27%) patients, while 77 (27%) patients had LPR (Table 2). The distribution of overall aggregation values (Figure 1A,B) and ADP‐induced aggregation was consistent across all three metabolizer groups (Figure 1C,D). Overall platelet aggregation was within the reference values when TEG was performed but was found to be rather low when assessed by MEA (Figure 1A,B). Aggregation values did not differ significantly between female and male patients across all metabolizer phenotypes (Figure S2).

**TABLE 2:** Ischemic and bleeding outcomes for patients with HPR and with LPR as assessed by TEG and/or MEA at 6 months ±2 weeks follow‐up.

Primary outcomes	HPR^a^				*p* Fisher	*p* Chi‐quadrat	OR	95%‐CI	*p* Logit. regression
	Yes (*n* = 76)		No (*n* = 207)						
MACE	9	(12%)	13	(6%)		0.136	2.005	0.820; 4.902	0.127
Death	5	(7%)	8	(4%)	0.345		1.752	0.555; 5.531	0.339
MI	2	(3%)	3	(1%)	0.613		1.838	0.301; 11.217	0.510
Stroke	2	(3%)	2	(1%)	0.293		2.770	0.383; 20.022	0.313

Secondary outcomes	LPR^a^				*p* Fisher	*p* Chi‐quadrat	OR	95%‐CI	*p* Logit. regression
	yes (*n* = 77)		no (*n* = 206)						
NMCR or major	13	(17%)	24	(12%)		0.242	1.540	0.740; 3.205	0.248
NMCR	4	(5%)	13	(6%)	1.000		0.813	0.257; 2.576	0.726
Major	10	(13%)	11	(5%)		0.040	2.646	1.075; 6.509	0.034
Any bleeding	35	(45%)	69	(33%)		0.072	1.655	0.970; 2.822	0.064
Minor bleeding	22	(29%)	45	(22%)		0.272	1.431	0.790; 2.594	0.237

*Note:* Values are in number and percentage, *n* (%).

Abbreviations: CI, confidence interval; HPR, high platelet reactivity; LPR, low platelet reactivity; MACE, major adverse cardiac events; Mi, myocardial infarction; NMCR, non‐major clinically relevant bleeding; OR, odds ratio.

^a^ HPR defined as upper quartile and LPR defined as lower quartile.

In patients with PM/IM status, the median of on clopidogrel PR detected by TEG was significantly higher compared with the rest of the patients (median MA_ADP_ 56.9 mm vs. 49.7 mm, *p* = 0.008) (Figure S1B). Notably, *CYP2C19* PM/IM status was associated with the presence of HPR status, as assessed by both the functional assays (OR 1.929, [confidence interval, 95% CI 1.088–3.420], *p* = 0.025) (Table 3).

**TABLE 3:** Association of metabolizer status with HPR and LPR.

HPR‐status (upper quartile > 17.5 U; > 58.1 mm)	PM/IM		NM + RM		*p* Chi‐quadrat	OR	95%‐CI	*p* Logit. regression
	*n* = 73 (%)		*n* = 210 (%)					
HPR	27	(37%)	49	(23%)	0.031	1.929	1.088; 3.420	0.025
No HPR	46	(63%)	161	(77%)	0.031			

LPR‐Status (lower quartile < 6.7 U; < 39.9 mm)	RM		PM/IM + NM		*p* Chi‐quadrat	OR	95%‐CI	*p* value Logit. regression
	*n* = 102 (%)		*n* = 181 (%)					
LPR	27	(26%)	50	(28%)	0.890	0.943	0.546; 1.631	0.834
No LPR	75	(74%)	131	(72%)	0.890			

*Note:* The values are in number and percentage, *n* (%).

Abbreviations: CI, confidence interval; HPR, high platelet reactivity; IM, intermediate metabolizer; LPR, low platelet reactivity; NM, normal metabolizer; OR, odds ratio; PM, poor metabolizer; RM, rapid metabolizer.

### 3.4 Association of *CYP2C19* Genotype With Clinical Outcomes

The primary composite ischemic outcome occurred in 22 (8%) patients. Overall, there were 13 (5%) all‐cause deaths, 5 (2%) myocardial infarctions and 4 (1%) strokes (Table 4). NMCR bleeding or major bleeding occurred in 37 (13%) patients, whereas minor bleeding occurred in 67 (24%) patients (Table 4 and Table S3). MACE incidence was notably higher in the first 30 days, particularly among patients with HPR or PM/IM status (Figure S3A,B). In contrast, NMCR or major bleeding events occurred consistently throughout the 6 months of follow‐up (Figure S3D,E).

**TABLE 4:** Ischemic and bleeding outcomes for poor/intermediate metabolizers versus rest of patients at 6 months ±2 weeks follow‐up.

	Events PM/IM (*n* = 73)		Events NM + RM (*n* = 210)		*p* Fisher	*p* Chi‐quadrat	OR	95%‐CI	*p* Logit. regression
	*n*	(%)	*n*	(%)					
Primary outcomes									
MACE	6	(8%)	16	(8%)		0.869	1.086	0.408; 2.889	0.869
Death	3	(4%)	10	(5%)	1.000		0.857	0.229; 3.204	0.819
Myocardial infarction	2	(3%)	3	(1%)	0.606		1.944	0.318; 11.869	0.472
Stroke	1	(1%)	3	(1%)	1.000		0.958	0.098; 9.360	0.971
Secondary outcomes									
NMCR or major	9	(12%)	28	(13%)		0.826	0.914	0.409; 2.041	0.826
NMCR	2	(3%)	15	(7%)	0.254		0.366	0.082; 1.642	0.189
Major	7	(10%)	14	(7%)		0.412	1.485	0.575; 3.836	0.414
Any bleeding	21	(29%)	83	(40%)		0.101	0.618	0.347; 1.01	0.102
Minor	12	(16%)	55	(26%)		0.091	0.554	0.278; 1.107	0.094

*Note:* The values are in number and percentage, *n* (%).

Abbreviations: CI, confidence interval; IM, intermediate metabolizer; MACE, major adverse cardiac events; NM, normal metabolizer; NMCR, non‐major clinically relevant bleeding; OR, odds ratio; PM, poor metabolizer; RM, rapid metabolizer.

PM/IM status did not associate with the primary composite ischemic outcome (OR 1.086, 95% CI [0.408–2.889], *p* = 0.869) (Table 4). Despite overall low rates, MI was more frequent in patients with PM/IM (3% vs. 1%, *p* = 0.606) (Table 4).

Even though not statistically significant, PM/IM phenotype indicated lower risk for NMCR bleeding (OR 0.366, 95% CI [0.082–1.642], *p* = 0.189) and minor bleeding (OR 0.554, 95% CI [0.278–1.107], *p* = 0.094) (Table 4). Any bleeding was less frequent in PM/IM (21 [29%]) compared to NM (42 [39%]) and RM (41 [40%]), *p* = 0.255 (Table S3). In summary, PM/IM phenotype might be an indicator for potentially reduced bleeding risk in this patient cohort.

RM phenotype associated neither with the ischemic risk (OR 0.645, 95% CI [0.244–1.703], *p* = 0.375), nor with the NMCR or major bleeding risk (OR 0.956, 95% CI [0.464–1.970], *p* = 0.902) (Table S4).

### 3.5 Association of Platelet Reactivity With the Outcomes

PR was assessed by TEG and/or MEA, with HPR defined as values in the upper quartile of overall measured values. HPR was not associated with increased ischemic risk 12% vs. 6%, *p* = 0.136; OR = 2.005 (95% CI [0.820–4.902], *p* = 0.127) (Table 2). However, there was a trend for increased MACE risk at 6 months (+/− 2 weeks) in patients with HPR as compared to those without HPR (hazard ratio, HR 2.25, *p* = 0.052) (Figure S3B).

LPR associated with significantly increased odds for major bleeding events (13% vs. 5% without LPR, *p* = 0.040), with an OR = 2.648 (95% CI 1.075–6.509, *p* = 0.034) (Table 2). Additionally, patients with LPR presented with almost 1.7‐fold increased odds for any bleeding events (Table 2).

Lastly, patients were grouped into phenotypes representing concordant metabolizer status and platelet reactivity. Accordingly, we grouped patients with both PM/IM and HPR and patients with RM and LPR. These phenotypes were compared with the rest of the patients and no increased risk for the occurrence of primary ischemic or secondary bleeding outcome was detected (Figure S3C,F).

## 4 Discussion

This study's main findings are: in patients with AF treated with an OAC and clopidogrel (1) there was no statistically significant association of PM/IM phenotype with the composite ischemic outcome at 6 months after PCI; (2) *CYP2C19* polymorphism of PM/IM indicated lower bleeding risk; (3) HPR might indicate a potential increase in ischemic events, while (4) patients with LPR were significantly more prone to major bleeding events; (5) HPR status was more frequent in patients with PM/IM phenotype.

### 4.1 Prevalence of *CYP2C19* Polymorphisms and Platelet Aggregation

Allele subtypes *2 and *17, in addition to the wild type *1, are the most common subtypes of the *CYP2C19* enzyme [9]. These alleles were investigated in this cohort due to their frequent occurrence in the Caucasian population, with CYP2C19*2 present in 15% and *17 in up to 21% [21]. When investigating the multi‐ethnic occurrence of different *CYP2C19* phenotypes, the proportion of ultrarapid metabolizers ranges from 2% to 5% [21]. The prevalence of phenotypes in this study's patient population, predominantly Caucasian, was consistent with the literature.

Notably, HPR rates detected by TEG and/or MEA in this study were considerably different from those reported in PCI patients treated with clopidogrel monotherapy, which range between 15% and 48% [4, 9]. Specifically, TEG detected HPR rates in PCI patients in literature range from 21% to 50% [5, 12]. The higher HPR rates observed in this study may be due to preanalytical factors such as duale ATT, older age (median 78 years), or increased intrinsic thromboembolic risk in this specific patient cohort with additional AF [22, 23]. Consistent with the guidelines for *CYP2C19* genotype and guided clopidogrel therapy [9], our analysis did not identify sex‐related differences in platelet function test responses across metabolizer groups, suggesting that sex is unlikely to significantly modify the CYP2C19–clopidogrel response genotype‐phenotype relationship in this cohort. Moreover, a significant proportion of patients might not be at a steady state concerning the antiplatelet effect of clopidogrel. While the two methods display different test principles [20] with TEG quantifying clot formation, strength and stability, MEA is based on platelet aggregation and might be more dependent on isolated platelet function. Given the discrepancies with literature on the detection of altered PR, we defined HPR as the upper quartile. These findings might indicate that the conventional cut‐off values in literature might be inappropriate and should be re‐evaluated for this specific cohort.

Lastly, the distribution of platelet aggregation across polymorphism subgroups was very similar, with HPR status being more frequent in poor metabolizers, suggesting a potential association between genotyping and functional assays as previously described in literature [24].

### 4.2 *CYP2C19* Polymorphism Association With Clinical Outcomes

The overall occurrence of the primary composite ischemic outcome and of the secondary bleeding outcome at 6 months follow‐up in this study cohort is consistent with the AUGUSTUS trial [25](6.5%–7.3%), one of the major RCTs including patients with AF undergoing PCI.

Previous studies have shown inconsistent results regarding the association of *CYP2C19* polymorphisms with bleeding events, whereas at 1 year after PCI, PM status associated with a 2 to 3‐fold increase of risk for ischemic event [24, 26]. In the TRITON‐TIMI study, carriers of at least one LOF *CYP2C19* allele had a 53% increased risk of the composite ischemic outcome (death, myocardial infarction, stroke) compared with non‐carriers, as well as a three‐fold increased risk for stent thrombosis [27]. In contrast, Soh et al. described a lack of association of metabolizer status with ischemic risk in patients undergoing PCI that received clopidogrel. Likewise, we did not detect a statistically significant association of metabolizer status with the ischemic outcomes. The lack of a significant association may be caused by the limited statistical power of the study as well as the competing risk of non‐cardiovascular death in the composite ischemic outcome. Another factor explaining the variation in success rates of genotype‐guided therapy in PCI patients and its inconsistent association with ischemic risk is the use of differing definitions of metabolizer status across studies [24, 26, 27, 28]. Of note, PM and IM status associate with lower level of active clopidogrel metabolite exposure and lower effect on platelet inhibition [29, 30], indicating that genotyping alone especially as a routine point‐of‐care bedside testing might not be sufficient to detect patients at ischemic risk.

Nevertheless, even though numerically very low, we showed that myocardial infarctions occurred more frequently in patients presenting with PM/IM phenotype.

One study could not detect any association between different *CYP2C19* polymorphisms and the occurrence of major or minor bleedings in patients undergoing PCI (HR 1.01) [28] but numerous studies suggest that RM status in patients with CAD can increase bleeding risk [6]. Findings from our analysis determined that PM/IM phenotype might protect from bleeding events in patients with AF undergoing PCI. Genotype‐guided antiplatelet therapy escalation from clopidogrel to ticagrelor or prasugrel in patients undergoing PCI was more successful for LOF‐carriers with lower MACE events and similar bleeding risk [31]. Even though, up to date, different studies show inconsistent results regarding the use of genotyping in cardiovascular patients, current consortium guidelines [9], as well as the expert consensus [4] support the use of genotype‐guided strategy to reduce the risk for major cardiovascular events without significantly increasing major bleeding risk in specific scenarios.

The findings from this study open up further discussion on the significance and usefulness of routine bedside point of care (POC) testing of *CYP2C19* genotype and platelet function. If confirmed, that in this cohort PM phenotype is not a risk factor for ischemic events, but rather offers protection against bleeding, it would be of interest to assess whether the current antithrombotic regimes in NM and RM might be too intensive and whether a de‐escalation approach could potentially reduce bleeding without increasing ischemic risk.

### 4.3 Platelet Reactivity and Association With Ischemic and Bleeding Outcomes

Multiple studies have demonstrated that clopidgrel‐mediated platelet inhibition is assay‐dependent. Various PFTs including light transmission aggregometry, MEA, VerifyNow P2Y12 frequently show only modest agreement and often classify patients differently, underlining inconsistencies between assays [32, 33]. However, the international consensus statement on platelet function testing explicitly states that the selection of assay depends largely on local site experience and availability, rather than on the superiority of one single test, and supports the use of MEA/Multiplate as a valid option for monitoring antiplatelet therapy in cardiovascular patients [10]. Assays used to assess PFT in this study are explained in detail in the methods section.

HPR status increases the risk for ischemic events and cardiovascular death in patients undergoing PCI without atrial fibrillation [34]. One study included 1608 patients undergoing PCI and assessed HPR status by MEA. Their findings suggested that HPR status was a risk factor for the occurrence of stent thrombosis (OR 9.4) [35].

On the other hand, LPR status associates with increased risk for bleedings in patients after PCI compared with non‐LPR patients (15.6% vs. 3.3%, *p* < 0.001) [36].

When PR is assessed by TEG, similar scenarios of HPR associating with increased ischemic risk and LPR with increased bleeding risk, respectively, are observed [34, 36].

This study confirmed that also in patients with AF treated with an OAC HPR status is an indicator for higher ischemic risk, whereas LPR is significantly associated with increased risk of major bleeding complications. Ultimately, these findings suggest that adding PFT to genotyping as a functional indicator of the aggregation process might be useful to better predict the clinical outcomes in this specific patient cohort.

### 4.4 Strengths and Limitations of the Study

The novelty of this study lies in being the first to explore the association between genotyping used to characterize levels of clopidogrel active metabolite, functional assays and the clinical complications in AF patients on oral anticoagulation undergoing PCI.

However, the lack of clopidogrel pharmacokinetic data limits the full interpretation of genotyping results, which taken alone might not completely predict ischemic risk in this cohort. Moreover, the small sample size limits the power to detect a significant association with ischemic events in this study. This was an observational study and the results are primarily hypothesis‐generating but could be the rationale for planning further randomized controlled studies.

Additionally, the homogeneous patient population, composed primarily of individuals of Caucasian descent as well as the lack of analysis for rare genotypes poses further challenges in generalizing the results. Nevertheless, an incorrect definition of genotyping seems rather unlikely due to the rarity of other variants within the Caucasian population. Information on concomitant medications was not obtained; given their potential to influence clopidogrel metabolism, we acknowledge this as a significant limitation of our study.

Lastly, the lack of a baseline and serial platelet aggregation measurements pose a further Iimitation to the results interpretation, whereas the pooling of patients from two separate cohorts with different PFT measurements (TEG and/or MEA) may have introduced inconsistencies.

## 5 Conclusion

In this pilot study of patients treated with both oral anticoagulation for atrial fibrillation and clopidogrel after PCI, there was no statistically significant association between CYP2C19*2 carriers of ≥ 1 loss of function allele (PM/IM status) and increased ischemic risk, whereas PM/IM status may confer a protective effect against bleeding events.

While HPR status as assessed by TEG and/or MEA might indicate a higher ischemic risk, LPR status was associated with significantly increased risk for major bleeding.

While the study was underpowered to detect stronger associations, these findings underscore the need for further research on the role of genetic testing and PFT in personalized medicine.

## Author Contributions

D.G. and C.B.O. wrote the manuscript and designed the research. V.A., H.V., S.S., D.H., C.J., and S.R. performed the research. V.A., H.V., S.S., T.L., S.L., D.T., D.D., D.W., and D.G. analyzed the data C.B.O. contributed new reagents/analytical tools.

## Funding

This work was supported by a grant from the Faculty of Medicine, University of Freiburg and Haemonetics (S.A. Signy‐Centre 1274 Signy‐Avenex, Switzerland to Christoph B. Olivier).

## Conflicts of Interest

C.B.O. reports research support from Haemonetics, Deutsche Forschungsgemeinschaft, Faculty of Medicine, Freiburg University; Else Kröner‐Fresenius Stiftung; honoraria: Bayer Vital GmbH, Daiichi Sankyo, Ferrer, Idorsia. D.G. reports research support by a grant from the German Research Foundation. D.T. reports research support by a grant from the Deutsche Herzstiftung. He received within the last 24 months consulting fees/payment for lectures including service on speakers' bureaus by Daiichi Sankyo and Eleva. All other authors declared no competing interests for this work.

## Supporting information


**Table S1:** Key inclusion and exclusion criteria.
**Table S2:** Periprocedural medication and medication at discharge.
**Table S3:** Primary and secondary outcomes at 6 months ±2 weeks follow‐up.
**Table S4:** Ischemic and bleeding outcomes in rapid metabolizers versus rest of patients at 6 months ±2 weeks follow‐up.
**Figure S1:** On clopidogrel platelet reactivity as assessed by MEA (A) and TEG (B) in patients with poor/Intermediate metabolizer status compared with the rest of the patients.
**Figure S2:** On clopidogrel platelet reactivity as assessed by TEG and MEA in (A) poor/intermediate, (B) normal and (C) ultrarapid/rapid metabolizers according to sex.
**Figure S3:** Cox regression analysis of the composite primary ischemic and secondary bleeding outcomes stratified by (A, D) metabolizer status, platelet reactivity (B, E) or combined phenotypes (C, F).
