# Supplementary material for: CYP2C19 Polymorphism and Platelet Aggregation‐Associated Risks in Atrial Fibrillation Patients Undergoing PCI
Source: Clin Transl Sci. 2026 May 19;19(6):e70598. doi: 10.1111/cts.70598 (PMC13185751; doi:10.1111/cts.70598)
Supplement: Supplementary file 1 — Table S1: Key inclusion and exclusion criteria. Table S2: Periprocedural medication and medication at discharge. Table S3: Primary and secondary outcomes at 6 months ±2 weeks follow‐up. Table S4: Ischemic and bleeding outcomes in rapid metabolizers versus rest of patients at 6 months ±2 weeks follow‐up. Figure S1: On clopidogrel platelet reactivity as assessed by MEA (A) and TEG (B) in patients with poor/Intermediate metabolizer status compared with the rest of the patients. Figure S2: On clopidogrel platelet reactivity as assessed by TEG and MEA in (A) poor/intermediate, (B) normal and (C) ultrarapid/rapid metabolizers according to sex. Figure S3: Cox regression analysis of the composite primary ischemic and secondary bleeding outcomes stratified by (A, D) metabolizer status, platelet reactivity (B, E) or combined phenotypes (C, F). [file CTS-19-e70598-s001.docx]

# **SUPPLEMENT**

## **Table S1. Key inclusion and exclusion criteria**

| **Inclusion criteria** |
| --- |
| - Informed consent - ≥18 years of age or older - Atrial fibrillation with an indication for oral anticoagulation (i.e. CHA2DS2VASC score ≥1 for males, ≥2 for females) - Percutaneous coronary intervention in the first 3 days |
| **Exclusion criteria** |
| - Contraindication to a DOAC (i.e. apixaban, dabigatran, edoxaban, rivaroxaban) or clopidogrel - History of stent-thrombosis - Uncompliant patient from the point of view of the principal investigator like for example.: assumed non-compliance, frequent use of alcohol and drugs or not willing to proceed according to the protocol (patient not willing to follow-up) - GPIIb/IIa inhibitor in the last 24h - Use of prasugrel or ticagrelor in the last 7 days |

**Table S2. Periprocedural medication and medication at discharge**

| **Medication** | | **Total**  **n=283** | | **PM/IM**  **n=73 26%** | | **NM**  **n=108 38%** | | **RM**  **n=102 32%** | | **p-value** |
| --- | --- | --- | --- | --- | --- | --- | --- | --- | --- | --- |
| **ASA** | |  |  |  |  |  |  |  |  |  |
|  | Loading | 231 | 82% | 62 | 231 | 82% | 82% | 80 | 78% | 0.530 |
|  | 250 mg loading | 86 | 30% | 22 | 86 | 30% | 31% | 30 | 29% | 0.947 |
|  | 300mg loading | 56 | 20% | 14 | 56 | 20% | 19% | 21 | 21% | 0.967 |
|  | 400mg loading | 71 | 25% | 21 | 71 | 25% | 27% | 21 | 21% | 0.406 |
|  | Maintenance therapy | 28 | 10% | 3 | 28 | 10% | 10% | 14 | 14% | 0.109 |
| **Clopidogrel** | |  |  |  |  |  |  |  |  |  |
|  | Loading | 221 | 78% | 55 | 221 | 78% | 81% | 78 | 76% | 0.548 |
|  | 300mg loading | 47 | 17% | 7 | 47 | 17% | 19% | 20 | 20% | 0.170 |
|  | 600mg loading | 174 | 61% | 48 | 174 | 61% | 63% | 58 | 57% | 0.454 |
|  | Maintenance therapy | 61 | 22% | 17 | 61 | 22% | 19% | 24 | 24% | 0.621 |
| **Bridging therapy** | |  |  |  |  |  |  |  |  |  |
|  | Enoxaparin/tinzaparin/UFH | 24 | 8% | 3 | 24 | 8% | 10% | 10 | 10% | 0.296 |
|  | OAC | 28 | 10% | 10 | 28 | 10% | 8% | 9 | 9% | 0.803 |
| **OAC at measurement** | | 232 | 82% | 62 | 85% | 232 | 82% | 81 | 79% | 0.638 |
| **OAC at discharge** | | 281 | 99% | 73 | 100% | 281 | 99% | 101 | 99% | 0.704 |
|  | Vitamin-K-antagonist | 8 | 3% | 3 | 8 | 3% | 2% | 3 | 3% | 0.665 |
|  | Edoxaban | 47 | 17% | 10 | 47 | 17% | 17% | 19 | 19% | 0.688 |
|  | Apixaban | 99 | 35% | 26 | 99 | 35% | 31% | 40 | 39% | 0.417 |
|  | Rivaroxaban | 115 | 41% | 32 | 115 | 41% | 44% | 35 | 34% | 0.266 |
|  | Dabigatran | 11 | 4% | 1 | 11 | 4% | 6% | 4 | 4% | 0.360 |
|  | *The values are n (%). Abbreviations: ASA acetylsalicylic acid, OAC oral anticoagulation, PM poor metabolizer, IM intermediate metabolizer, NM normal metabolizer, RM rapid metabolizer, UFH unfractionated heparin.* | | | | | | | | | |

**Table S3. Primary and secondary outcomes at 6 months ± 2 weeks follow-up**

| **Outcomes** | **Total**  **n=283** | | **PM/IM**  **n=73 26%** | | **NM**  **n=108 38%** | | **RM**  **n=102 36%** | | **p-value**  **Fisher** | **p-value**  **Chi-quadrat** |
| --- | --- | --- | --- | --- | --- | --- | --- | --- | --- | --- |
| **Primary outcomes** |  |  |  |  |  |  |  |  |  |  |
| MACE | 22 | 8% | 6 | 8% | 10 | 9% | 6 | 6% |  | 0.650 |
| Death | 13 | 5% | 3 | 4% | 6 | 6% | 4 | 4% | 0.879 |  |
| Myocardial infarction | 5 | 2% | 2 | 3% | 1 | 1% | 2 | 2% | 0.641 |  |
| Stroke | 4 | 1% | 1 | 1% | 3 | 3% | 0 | 0% | 0.247 |  |
| **Secondary outcomes** |  | |  | |  | |  | |  |  |
| NMCR or major | 37 | 6% | 9 | 12% | 15 | 14% | 13 | 13% |  | 0.947 |
| NMCR | 17 | 6% | 2 | 3% | 9 | 8% | 6 | 6% | 0.318 |  |
| Major | 21 | 7% | 7 | 10% | 6 | 6% | 8 | 8% |  | 0.585 |
| Any bleedings | 104 | 37% | 21 | 29% | 42 | 39% | 41 | 40% |  | 0.255 |
| Minor bleedings | 67 | 24% | 12 | 16% | 27 | 25% | 28 | 28% |  | 0.220 |
| *The values are in number and percentage, n (%). Abbreviations: MACE major adverse cardiac events, NMCR non-major clinically relevant, PM poor metabolizer, IM intermediate metabolizer, NM normal metabolizer, RM rapid metabolizer* | | | | | | | | | | |

**Table S4. Ischemic and bleeding outcomes in rapid metabolizers vs. rest of patients at 6 months ± 2 weeks follow-up**

| **Primary outcomes** | **Events**  **RM (n=102)** | | **Events**  **NM + PM/IM (n=181)** | | **p-value**  **Fisher** | **p-value Chi- Quadrat** | **OR** | **95%-CI** | **p-value Logit. Regression** |
| --- | --- | --- | --- | --- | --- | --- | --- | --- | --- |
|  | n | (%) | n | (%) |  |  |  |  |  |
| MACE | 6 | (6%) | 16 | (9%) |  | 0.372 | 0.645 | 0.244; 1.703 | 0.375 |
| Death | 4 | (4%) | 9 | (5%) | 0.776 |  | 0.780 | 0.234; 2.599 | 0.686 |
| Myocardial infarction | 2 | (2%) | 3 | (2%) | 1.000 |  | 1.187 | 0.195; 7.221 | 0.853 |
| Stroke | 0 | (0%) | 4 | (2%) | 0.300 |  | 0.00 | - |  |
| **Secondary outcomes** | **Events**  **RM (n=102)** | | **Events**  **NM + PM/IM (n=181)** | | **p-value**  **Fisher** | **p-value Chi- Quadrat** | **OR** | **95%-CI** | **p-value Logit. Regression** |
|  | n | (%) | n | (%) |  |  |  |  |  |
| NMCR or major | 13 | (13%) | 24 | (13%) |  | 0.902 | 0.956 | 0.464; 1.970 | 0.902 |
| NMCR | 6 | (6%) | 11 | (6%) |  | 0.947 | 0.966 | 0.346; 2.694 | 0.947 |
| Major | 8 | (8%) | 13 | (7%) |  | 0.839 | 1.099 | 0.440; 2.749 | 0.839 |
| Any bleeding | 41 | (40%) | 63 | (35%) |  | 0.367 | 1.259 | 0.763; 2.076 | 0.367 |
| Minor | 28 | (28%) | 39 | (22%) |  | 0.262 | 1.378 | 0.786; 2.414 | 0.263 |
| *The values are in number and percentage, n (%). Abbreviations: MACE major adverse cardiac events, NMCR non-major clinically relevant bleeding, PM poor metabolizer, IM intermediate metabolizer, NM normal metabolizer, RM rapid metabolizer, OR odds ratio, CI confidence interval.* | | | | | | | | | |

**Figure S1. On clopidogrel platelet reactivity as assessed by MEA (A) and TEG (B) in patients with poor /Intermediate metabolizer status compared with the rest of the patients**

**(A)**

**
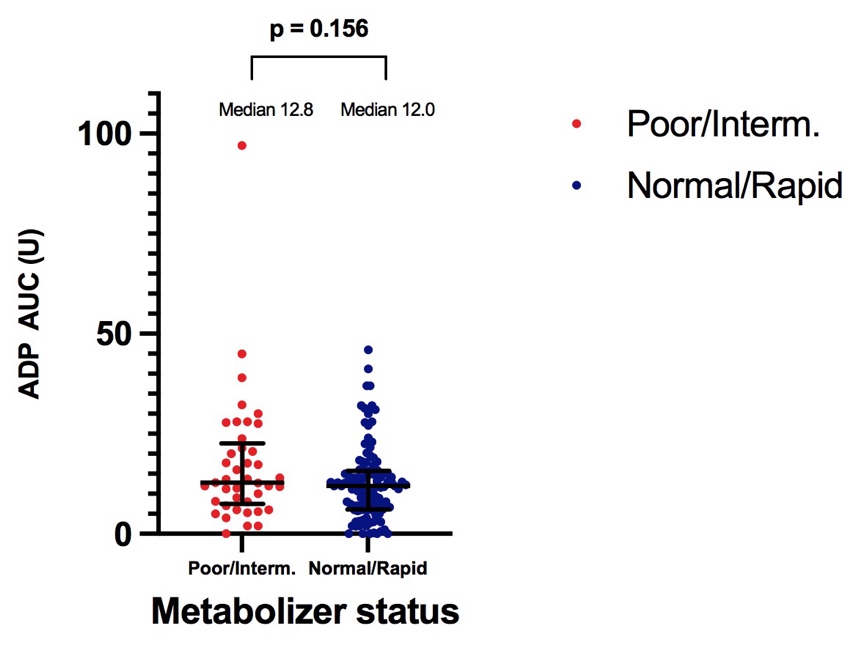
**

**(B)**

**
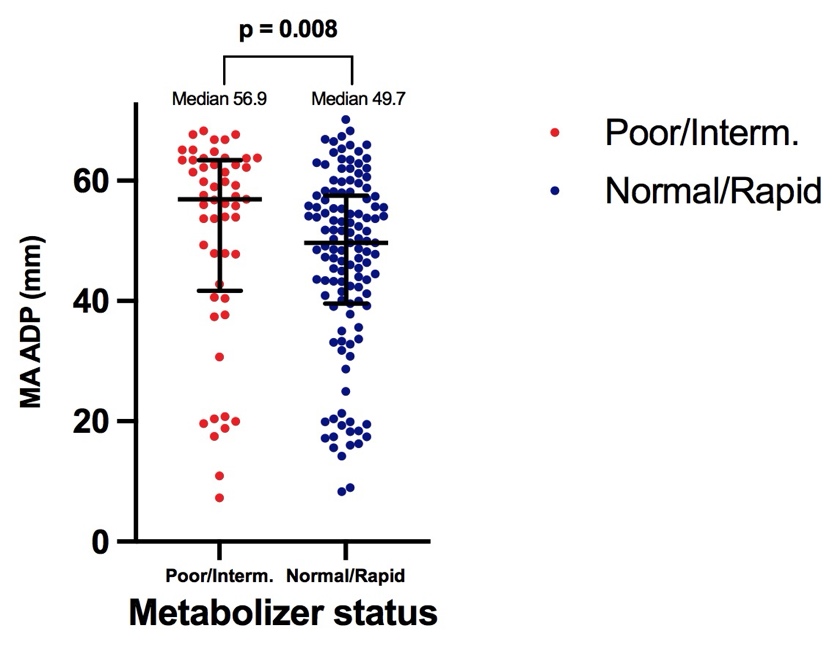
**

*Abbreviations: ADP AUC adenosine diphosphate–area under the curve, MA maximal amplitude.*

**Figure S2. On clopidogrel platelet reactivity as assessed by TEG and MEA in (A) poor/intermediate, (B) normal and (C) ultrarapid/rapid metabolizers according to sex.**

**(A)**

**(B)**

**
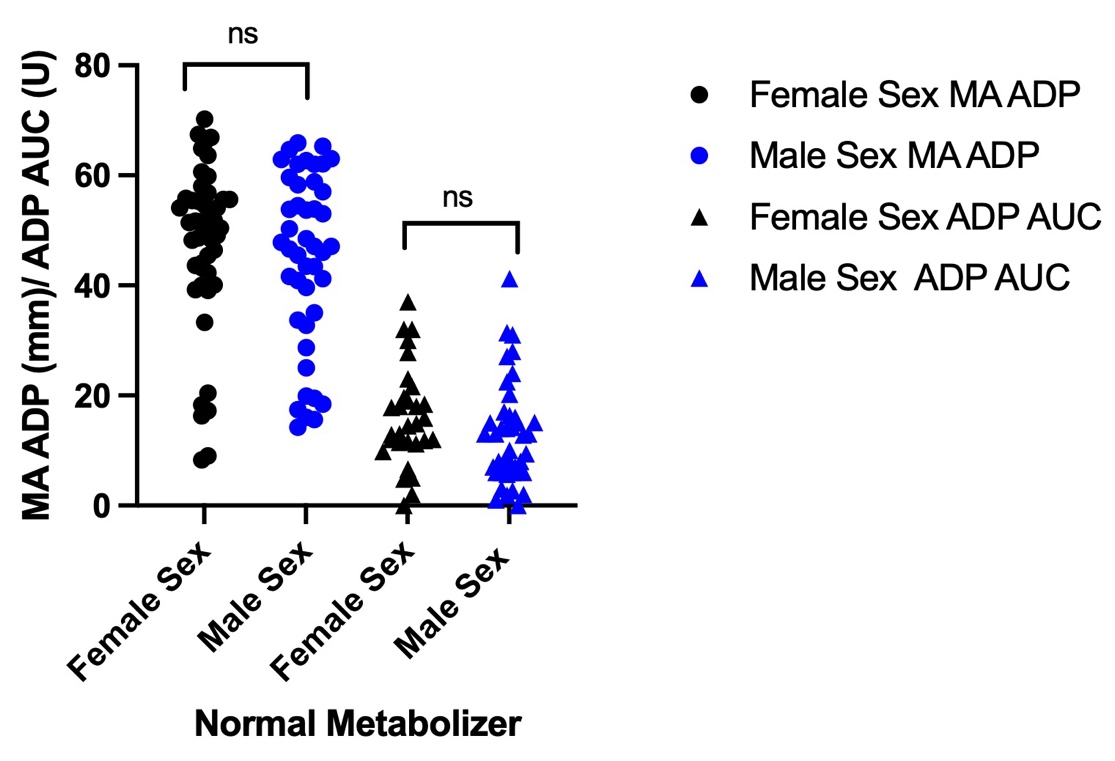
**

**(C)**

*Abbreviations: ADP AUC adenosine diphosphate–area under the curve, MA maximal amplitude.*

## **Figure S3. Cox regression analysis of the composite primary ischemic and secondary bleeding outcomes stratified by (A, D) metabolizer status, platelet reactivity (B, E) or combined phenotypes (C, F)**

**(A)**


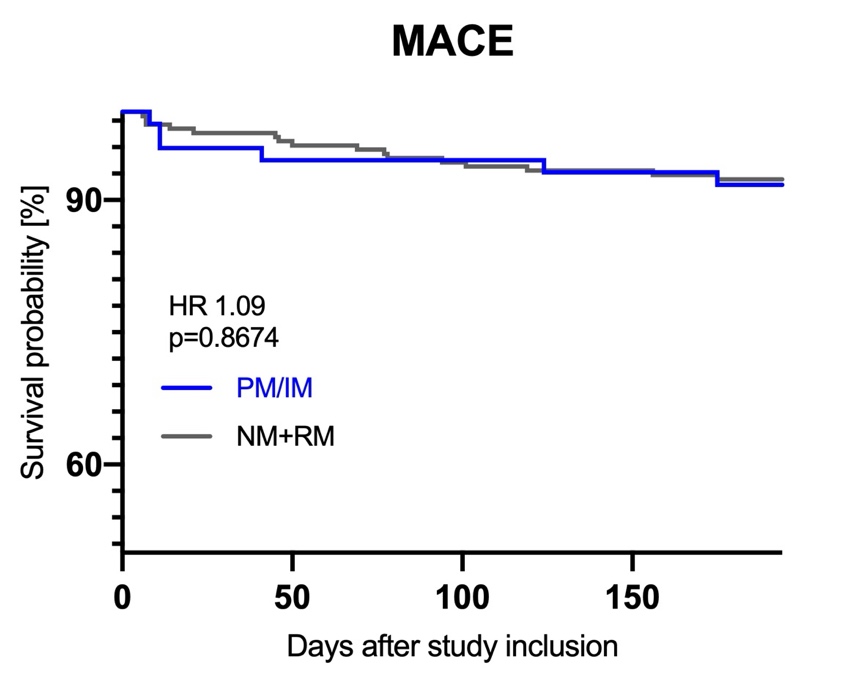


**(B)**


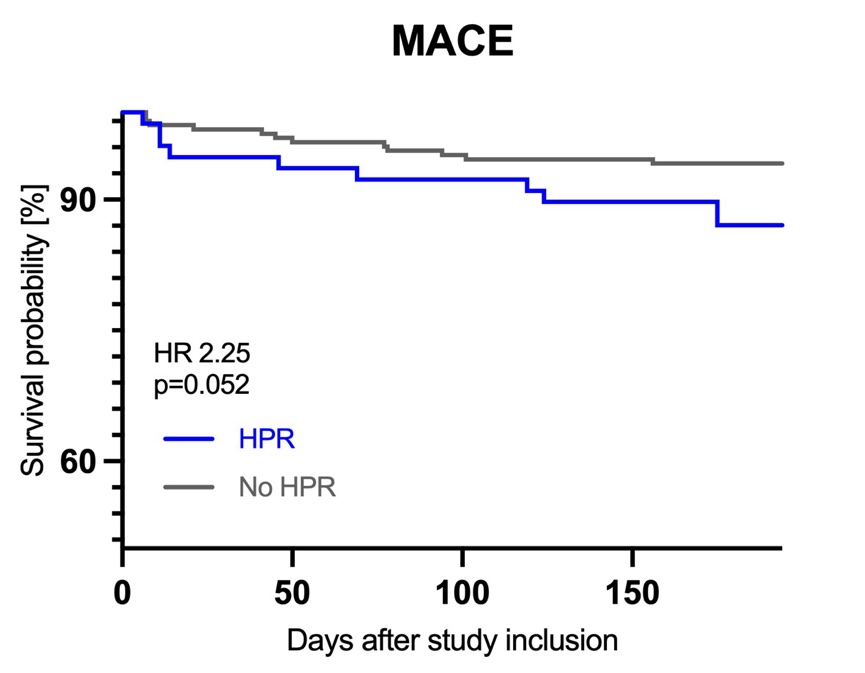


**(C)**

**(D)**


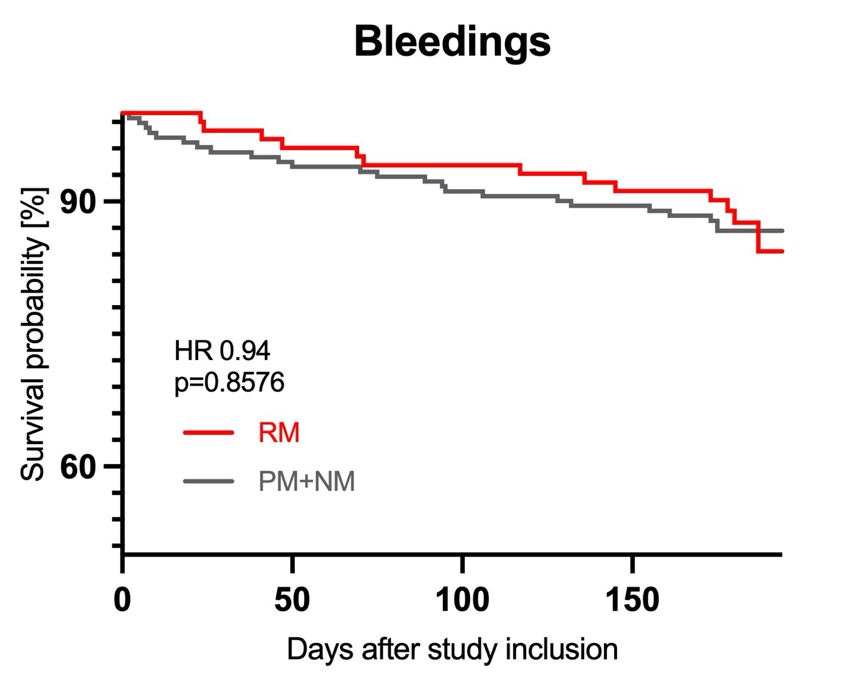


**(E)**


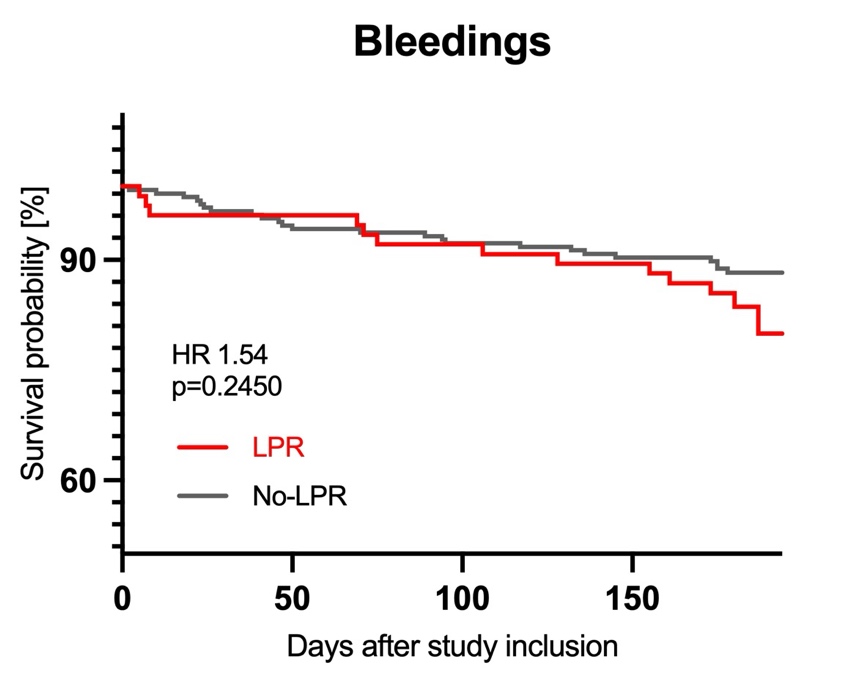


**(F)**

*Abbreviations: MACE major adverse cardiac events, PM poor metabolizer, NM normal metabolizer, RM rapid metabolizer, HR hazard ratio, HPR high platelet reactivity, LPR low platelet reactivity.*
